# Diphtheria

**Published:** 1879-04

**Authors:** D. H. Chase

**Affiliations:** Louisville


DIPHTHERIA.—*Editor Medical Brief:* In the past three years I have treated nearly two hundred cases of diphtheria, as follows:

| | | |
|---|---|---|
| ℞.—Tr. chloride iron, | . . . . | 1 oz. |
| Potas. chlorate, | . . . . | 1 dchm. |

Mix. Sig. Give five to twenty drops in a swallow of water every four hours, and if throat is badly affected, use a swab of the pure tincture of iron and glycerine, once daily for three days.

For fever:

| | | |
|---|---|---|
| ℞.—Tr. aconite, | . . . | 10 drops. |
| Tr. gelseminum, | . . . | 20 drops. |
| Tr. phytolacca, | . . . | 20 drops. |
| Water, | . . . . | 4 ounces. |

Mix. Sig. One teaspoonful every hour.

With this treatment I have not lost a case.

D. H. CHASE, M.D.

*Louisville, March* 4, 1879.

